# Fabry Disease: A Focus on the Role of Oxidative Stress

**DOI:** 10.3390/antiox15020168

**Published:** 2026-01-26

**Authors:** Julia Rydzek, Adrian Muzyka, Krzysztof Majcherczyk, Julia Soczyńska, Wiktor Gawełczyk, Mateusz Żołyniak, Sławomir Woźniak

**Affiliations:** 1 Student Scientific Society Anatomia-Klinika Nauka, Division of Anatomy, Department of Human Morphology and Embryology, Wroclaw Medical University, 50-367 Wroclaw, Poland; adrian.muzyka@student.umw.edu.pl (A.M.); krzysztof.majcherczyk@student.umw.edu.pl (K.M.); julia.niznik@student.umw.edu.pl (J.S.); wiktor.gawelczyk@student.umw.edu.pl (W.G.); mateusz.zolyniak@student.umw.edu.pl (M.Ż.); 2 Division of Anatomy, Department of Human Morphology and Embryology, Wroclaw Medical University, 50-367 Wroclaw, Poland

**Keywords:** Fabry disease, oxidative stress, reactive oxygen species, biomarkers, organ involvement

## Abstract

Fabry disease is an X-linked lysosomal storage disorder caused by mutations in the *GLA* gene, leading to α-galactosidase A deficiency, accumulation of globotriaosylceramide (Gb3), and progressive multiorgan involvement. Increasing evidence indicates that oxidative stress plays a central role in disease pathogenesis. This review aims to synthesize current knowledge on the molecular mechanisms underlying oxidative stress, the relevance of oxidative damage biomarkers, and potential therapeutic implications. A comprehensive literature search was conducted in PubMed/MEDLINE, Scopus, Web of Science, and Google Scholar using terms related to Fabry disease, Gb3 metabolism, mitochondrial and endothelial dysfunction, inflammatory signaling, and oxidative stress markers. Clinical, experimental, and translational studies were included. Available data demonstrate that Gb3 accumulation disrupts mitochondrial function and activates NADPH oxidase, NF-κB, and MAPK signaling pathways, resulting in excessive production of reactive oxygen species. These processes contribute to cellular injury, particularly within the cardiovascular, renal, and nervous systems. Biomarkers such as malondialdehyde, 8-hydroxy-2′-deoxyguanosine, glutathione redox status, and antioxidant enzyme activities appear useful for assessing oxidative burden and monitoring therapeutic responses. Overall, current evidence underscores the pivotal role of oxidative stress in the progression of Fabry disease and highlights the need for further research into targeted antioxidant and disease-modifying therapeutic strategies.

## 1. Introduction

Fabry disease (FD) is a rare, X-linked lysosomal storage disorder caused by pathogenic variants in the *GLA* gene, resulting in deficiency of α-galactosidase A [1]. Impaired enzymatic activity leads to progressive intracellular accumulation of globotriaosylceramide (Gb3) and its deacylated derivative globotriaosylsphingosine (lyso-Gb3) in multiple cell types, including endothelial cells, cardiomyocytes, podocytes, neurons, and smooth muscle cells [1]. This widespread cellular involvement underlies the characteristic multisystem manifestations of Fabry disease, affecting the cardiovascular, renal, nervous, and vascular systems [2].

While lysosomal glycosphingolipid accumulation represents the primary biochemical defect in Fabry disease, it initiates a series of downstream pathological processes that contribute to progressive organ damage [3]. Among these secondary mechanisms, oxidative stress has attracted increasing attention due to its consistent presence across different tissues and disease stages [4]. Experimental and clinical studies demonstrate that excess Gb3 and lyso-Gb3 disrupt cellular redox balance by increasing the generation of reactive oxygen species (ROS), impairing nitric oxide bioavailability, and weakening endogenous antioxidant defense systems [4]. Importantly, these alterations are observed not only in advanced disease but also in early stages and persist in many patients despite enzyme replacement therapy, indicating that oxidative stress may amplify and perpetuate tissue injury rather than merely reflect terminal organ failure [5].

At the molecular level, glycosphingolipid storage-induced oxidative stress involves multiple interconnected pathways [6]. Mitochondrial dysfunction, impaired autophagy, activation of NADPH oxidases, uncoupling of nitric oxide synthase, and dysregulation of antioxidant enzymes collectively contribute to sustained redox imbalance [6,7]. These processes activate redox-sensitive inflammatory and profibrotic signaling cascades, including NF-κB, MAPK, and TGF-β pathways, thereby linking oxidative stress to endothelial dysfunction, vascular remodeling, myocardial hypertrophy, renal fibrosis, and progressive loss of organ function [7]. Consistently, increased levels of oxidative stress biomarkers—such as malondialdehyde, 8-hydroxy-2′-deoxyguanosine, advanced oxidation protein products, and nitrotyrosine—have been reported in both untreated and treated patients with Fabry disease [8].

Clinically, oxidative stress is associated with major organ-specific complications of Fabry disease [1]. In the cardiovascular system, redox imbalance contributes to left ventricular hypertrophy, myocardial fibrosis, arrhythmogenesis, and microvascular dysfunction [9]. In the kidneys, oxidative stress accompanies podocyte injury, proteinuria, and progressive nephropathy [9]. In the nervous system, it is implicated in neuropathic pain and cerebrovascular pathology [10]. Although current disease-specific therapies effectively reduce substrate accumulation, they do not fully normalize redox homeostasis, which may help explain the continued progression of organ damage in some patients [11].

The aim of this review is to provide a focused overview of oxidative stress as a secondary but clinically relevant mechanism in Fabry disease. We summarize molecular sources of ROS, redox-dependent signaling pathways, and disturbances of antioxidant defenses, and relate these processes to organ-specific clinical manifestations. In addition, we discuss established and emerging biomarkers of oxidative stress and review current and experimental therapeutic strategies, with particular emphasis on redox-modulating approaches as adjuncts to enzyme- and chaperone-based therapies. By integrating mechanistic and clinical evidence, this review highlights oxidative stress as an important modifier of disease progression and a potential target for complementary therapeutic interventions in Fabry disease.

**Figure 1:**
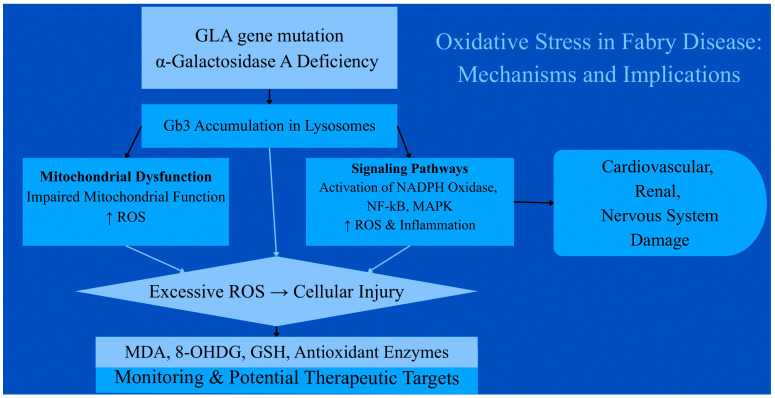
The Elements Discussed in the Text.

Figure 1 presents the key aspects that will be discussed in this review.

## 2. Molecular Mechanisms of Oxidative Stress in Fabry Disease

Physiologically, α-galactosidase A mediates one of the stages of the degradation process of Gb3 in lysosomes: the removal of terminal α-galactose residues to yield lactosylceramide. Deficiency of α-galactosidase A leads to lysosomal accumulation of the compound in various cell types [12].

Gb3 is considered the main storage material; however, in patients with Fabry disease, accumulation of the deacylated form, lyso-Gb3, as well as galabiosylceramides (Gb2) and blood group antigens B, B1, and P1—all containing a terminal α-galactose residue—can also be observed [3,13]. There are certain differences among these compounds that encompass not only structure but also the site of accumulation in the disease. Moreover, structure may influence the aforementioned localization. For example, Gb3 is characterized by a more hydrophobic nature than lyso-Gb3; therefore, its high concentration is observed in organs. A hypothesis has been proposed regarding the production of lyso-Gb3 as a strategy to prevent Gb3 accumulation in α-galactosidase A deficiency [14]. However, we have decided to describe the details of tissue localization in another paragraph. In the present section, we instead focus on biological activity.

Gb3 accumulated in lysosomes—which are responsible for cellular clearance, energy balance, and the metabolism and transport of certain compounds—according to the literature, initiates a cascade of disturbances associated with the persistence of damaged organelles and protein aggregates, leading to the development of inflammation and the generation of oxidative stress [1,15]. Lyso-Gb3 also contributes to the propagation of inflammation, *inter alia* through an autophagy-dependent necroptosis pathway [16]. The literature focuses on these latter aspects with regard to pro-oxidative effects. Below, we describe the most important mechanisms associated with the disruption of redox homeostasis in FD.

### 2.1. Mitochondrial Dysfunction as a Source of Reactive Oxygen Species

Mitochondrial disturbances in FD are associated, *inter alia*, with impairments in autophagy. A mechanism has been proposed involving the dysfunction of mTOR, which regulates autophagy, in response to the accumulation of sphingolipids in lysosomes [1,17]. Accumulating pathological mitochondria are a source of mitochondrial DNA and reactive oxygen species; when released into the cytosol, these factors trigger the activation of the NLRP3 inflammasome and the secretion of cytokines such as IL-1β or IL-18 [1]. Activation of the inflammasome, in turn, is associated with subsequent mitochondrial dysfunction, including, *inter alia*, depolarization of the mitochondrial membrane [18].

Moreover, regarding other disturbances of these organelles, authors have described reduced metabolism and decreased activity of respiratory chain complexes I, IV, and V in fibroblast cultures from FD patients, as well as decreased levels of AMP, ADP, and ATP [19]. In parallel, disturbances in the regulation of mitochondrial microRNAs (miRs) are observed. For instance, miR-1, which performs protective functions for mitochondria, was found to be downregulated in FD patients [20]. This loss of regulatory oversight appears to exacerbate organelle dysfunction. The energy deficit is further intensified by impaired mitochondrial β-oxidation. This is further aggravated by the inhibition of autophagy, which creates difficulties in the transport of fatty acids from lipid droplets to mitochondria [21]. The accumulation of mitochondrial disturbances and the production of reactive oxygen species may be further amplified by the phenomenon of ROS-induced ROS release, leading to DNA damage and apoptosis [22].

### 2.2. Non-Mitochondrial Sources of Reactive Oxygen Species

ROS production in cells is, to some extent, dependent on membrane-bound NADPH oxidases (NOX). Their activity is based on the cooperation of membrane-bound and cytoplasmic subunits. In the context of FD, particular attention is paid to the NOX2 isoform, whose activity requires association with p22phox alongside three other subunits; this results in the transfer of electrons from NADPH to molecular oxygen and the generation of the superoxide anion, initiating the release of subsequent ROS. Ravarotto et al. reported an overexpression of p22phox in FD patients, which was accompanied by intensified lipid peroxidation [5].

The literature contains references to cross-talk between mitochondrial-derived ROS and other sources, such as the aforementioned NOX [23]. The presence of NOX-derived ROS is associated with enhanced production of mitochondrial ROS [24]. Another study indicates the ability of mitochondrial ROS to activate phagocytic and cardiovascular NOX [25], suggesting the possible existence of positive feedback between mitochondrial and non-mitochondrial sources of ROS, leading to self-perpetuating oxidative stress.

Importantly, Gb3 storage leads to the activation of Toll-like receptors (TLRs), such as TLR4, resulting in the production of proinflammatory cytokines and chemokines. Lyso-Gb3 bound to TLR4, through the activation of NOTCH1, also leads to the production of these cytokines indirectly via the nuclear factor kappa B (NF-κB) pathway [26]. Through NF-κB, TLR4 has the ability to enhance inducible Nitric Oxide Synthase (iNOS) expression [27]. iNOS is capable of producing the superoxide anion, which, in reaction with NO, forms peroxynitrite, further contributing to oxidative stress [28]. Furthermore, in response to the loss of α-galactosidase A activity in human endothelial cell cultures, increased levels of 3-nitrotyrosine (a marker of oxidative stress) and decreased endothelial Nitric Oxide Synthase (eNOS) activity have been demonstrated. This appears to be confirmed by a study of plasma samples from patients with the classical FD phenotype, which showed increased concentrations of 3-nitrotyrosine [29]. It is worth noting that, as a consequence of the above, NO bioavailability decreases [4].

Interestingly, there are suggestions regarding direct interactions between TLR4 and NOX. Further mechanisms associated with this, as mentioned earlier, include electron transport across the membrane and subsequent ROS generation [27].

**Figure 2:**
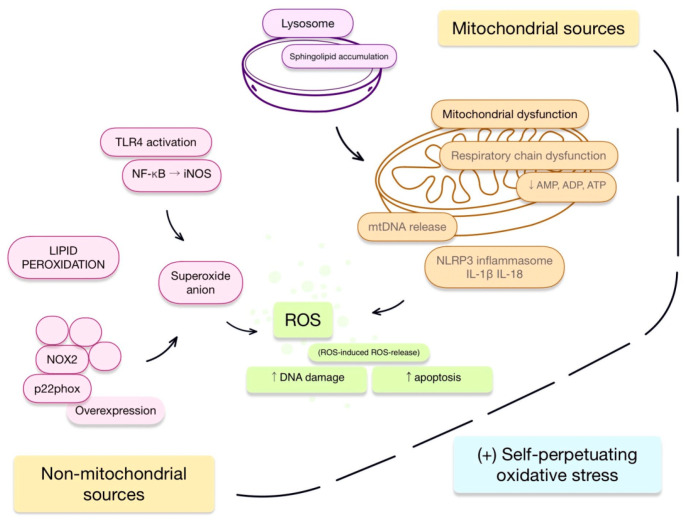
Proposed mechanisms of reactive oxygen species production in Fabry disease.

Figure 2 presents a summary of the proposed mechanisms discussed in the last two paragraphs.

### 2.3. Dysregulation of Antioxidant Defense Systems

Oxidative stress results not only from the increased production of oxidants but also from reduced antioxidant capacity [23]; indeed, FD patients are characterized by decreased antioxidant resistance [30]. Key antioxidant enzymes include superoxide dismutase (SOD), glutathione peroxidase, and catalase (CAT) [31]. The literature reports findings regarding both the expression and activity of these enzymes, which are summarized below.

SOD2 is a mitochondrial matrix protein responsible for protecting the cell against ROS. A study of endothelial cells derived from FD patients demonstrated that Gb3 accumulation is associated with decreased SOD2 expression [32]. The probable mechanism of the latter is described later in this review. CAT, which participates in the decomposition of hydrogen peroxide into water and oxygen [33], has also been indirectly analyzed in the context of Fabry disease. The results of studies measuring its activity, however, are inconsistent.

In a study by Biancini et al., an increased SOD/CAT activity ratio was observed. Catalase activity was determined based on the rate of decrease in hydrogen peroxide absorbance at 240 nm (using the method described by Aebi), whereas SOD activity was measured using the RANSOD^®^ kit (Randox Lab, Antrim, UK) [34]. In contrast, a study by Müller et al. demonstrated an increase in CAT activity (method described by Adamo et al.) without significant changes in SOD (method described by McCord and Fridovich) compared to the control group. These discrepancies may be attributed to differences in methodology and population characteristics [35,36,37].

Another analyzed enzyme, glutathione peroxidase, increases the antioxidant potential of glutathione and is primarily responsible for the detoxification of peroxides [38]. In FD, reduced activity of glutathione peroxidase is observed alongside increased levels of reduced glutathione [39]. Furthermore, an increase in Gb3 levels is associated with reduced amounts of tetrahydrobiopterin (BH4), a necessary NOS cofactor. This leads to the risk of eNOS uncoupling, which is associated with superoxide anion production and a further reduction in antioxidant capacity [40].

### 2.4. Activation of Redox-Dependent Signaling Pathways in Inflammation

In Fabry disease (FD), early accumulation of Gb3 and lyso-Gb3 in the endothelium and cardiomyocytes activates TLR4-dependent signaling, triggering oxidative stress (ROS) and pro-fibrotic responses before typical inclusion bodies appear [4,41,42]. Misfolded α-GalA further induces endoplasmic reticulum stress and the unfolded protein response (UPR, or ‘agalopathy’), which independently drives inflammatory and fibrotic processes [1,43,44]. Excess ROS disrupt the balance between pro- and antioxidant mechanisms, damaging lipids, proteins, and DNA, while activating transcription factors such as NF-κB [45]. This leads to upregulation of proinflammatory cytokines (IL-1, IL-6, IL-12, TNF-α), chemokines, adhesion molecules, and enzymes including iNOS and cyclooxygenase-2, creating a feedback loop that amplifies oxidative stress [4,46]. Concurrently, MAPK pathways, particularly p38 MAPK, enhance the expression of inflammatory mediators, may suppress SOD activity, and induce α-smooth muscle actin (α-SMA), promoting fibrosis [47]. These interconnected processes demonstrate that in FD, inflammatory and fibrotic pathways are initiated early, driven both by substrate accumulation and stress from misfolded α-GalA, independently of visible lysosomal inclusions [1,47].

### 2.5. Endothelial Dysfunction and Fibrotic Remodeling as Consequences of Oxidative Stress

FD patients are characterized by increased levels of proinflammatory cytokines and the presence of oxidative damage [48]. In light of the described mechanisms, endothelial cells are affected, exhibiting, for example, higher levels of Intercellular Adhesion Molecule-1 (ICAM-1) and Vascular Cell Adhesion Molecule-1 (VCAM-1), as well as vascular smooth muscle involvement, resulting in remodeling and stiffness [4]. With regard to the kidneys, inflammation and the presence of activated immune cells promote the migration of myofibroblasts, their activation, and fibrosis. The aforementioned α-SMA is their marker. In FD patients, TGF-β is produced by proximal tubular cells, further inducing fibrosis through activation of myofibroblasts in vessels and glomeruli [48]. Alongside TGF-β, the effect of Gb3 on endothelial cells also causes expression of Vascular Endothelial Growth Factor Receptor 2 (VEGFR2), Vascular Endothelial Growth Factor alpha (VEGFα), and Fibroblast Growth Factor 2 (FGF2), which exert angiogenic effects [49]. Clinical consequences of the above have been addressed in a separate paragraph.

## 3. Biomarkers of Oxidative Stress

After describing the molecular mechanisms, we now move on to biomarkers of oxidative stress in Fabry disease. In recent years, scientists have focused on explaining the role of oxidative stress in the progression of the disease. An important study in this theory was the 2012 study by Biancini et al. [34]. The researchers showed that Fabry disease is characterized by pro-oxidative states in the form of reduced antioxidant defense mechanisms and high concentrations of malondialdehyde (MDA) [34]. Due to the key role of oxidative stress in the pathophysiology of many diseases, there is growing interest in the use of oxidative stress biomarkers in treatment monitoring [50]. The literature also points to elevated levels of oxidative stress markers in untreated patients with FD-specific mutations and normal Gb3 concentrations. These results indicate that oxidative stress markers can be used in the early diagnosis of the disease [4]. However, the usefulness of these biomarkers remains at the preclinical and clinical stages in a limited number of patients. Validation studies are necessary to exploit the potential of biomarkers [4,8,9,30,51].

**Figure 3:**
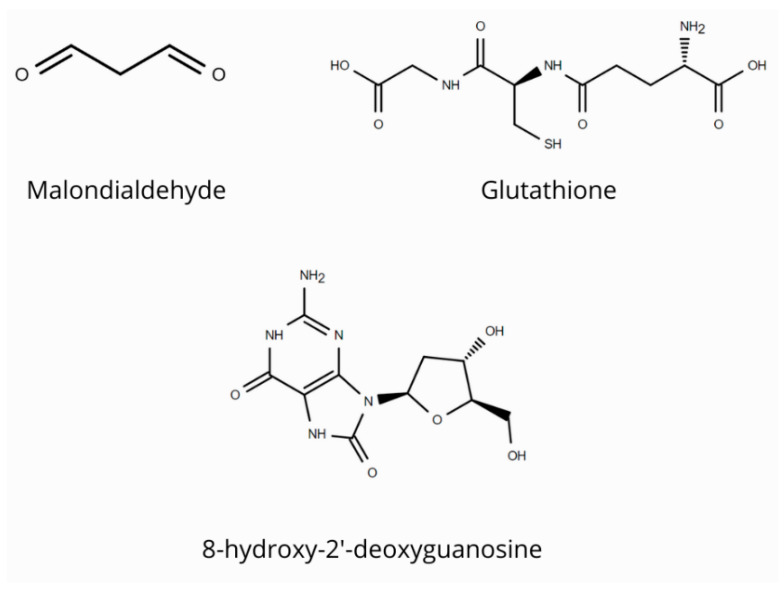
Structures of selected oxidative stress biomarkers.

The purpose of this section is to discuss the most important biomarkers of oxidative stress in FD, whose structures are shown in Figure 3.

### 3.1. Malondialdehyde

MDA is one of the most commonly studied biomarkers of oxidative stress. MDA is formed as a result of the peroxidation of polyunsaturated fatty acids in the lipid bilayer of cells, caused by ROS [52,53,54]. In a study by Ravarotto et al., higher plasma MDA levels were observed in FD patients (54.51 nmol/L) compared to healthy individuals (30.05 nmol/L) [5,55]. Despite satisfactory plasma results, studies of urine MDA levels did not show a statistically significant difference in MDA concentrations between FD patients and control group patients. This study indicates the limited usefulness of urine MDA detection in monitoring oxidative stress in FD [56].

### 3.2. 8-OHdG

Another marker used in FD monitoring is 8-hydroxy-2′-deoxyguanosine (8-OHdG). 8-OHdG is formed as a result of DNA oxidation by free radicals [6]. The pathophysiology of 8-OHdG formation involves the attack of hydroxyl radicals on guanine bases in DNA. The modified nucleotides are then removed by repair mechanisms and released into the peripheral circulation [57,58].

A study by Chen et al. showed that patients with cardiomyopathy in Fabry disease had higher 8-OHdG concentrations (4.5 ng/mL) than patients in the control group (3.4 ng/mL). After 14 months of enzyme replacement therapy (ERT), it was shown that in patients with cardiomyopathy, the concentration of 8-OHdG decreased to 4 ng/mL. The researchers emphasize that the concentration of 8-OHdG is clinically significant both as a non-invasive biomarker and as a parameter in monitoring patients undergoing ERT [59]. In another study by Chimenti et al. on cardiomyocyte dysfunction in FD, the presence of 8-OHdG was demonstrated in 25% of cardiomyocyte nuclei, with no 8-OHdG in control samples. The researchers showed that cardiomyocyte apoptosis was 187 times more frequent in FD patients than in the control group, and 8-OHdG was found in the nuclei of apoptotic cells [7].

### 3.3. Glutathione

Glutathione is described as one of the most important antioxidants in the human body. Glutathione protects cells from oxidative stress by neutralizing ROS [60]. The study by Biancini et al. showed elevated GSH levels in patients with FD at the time of diagnosis [61]. The oxidized form of glutathione—glutathione disulfide (GSSG)—can also be used as a marker. Studies by Schumann et al. showed a 2.2-fold increase in GSSG concentration in the kidney cells of patients with FD compared to the control group and a decrease in the GSH-to-GSSG ratio, confirming the consumption of glutathione in antioxidant mechanisms [62].

### 3.4. Other Biomarkers

Other biomarkers include antioxidant enzymes, which are important factors in removing ROS. These include, among others, superoxide dismutase (SOD) and catalase [32]. In a study by Tseng et al., vascular endothelial cells derived from iPSCs that reproduced the FD phenotype were examined. The study showed that Gb3 accumulation inhibits SOD2 expression, increasing ROS production [32]. In patients with FD, an increased SOD to catalase ratio is also observed in erythrocytes [7]. The literature indicates that in FD patients, increased SOD to catalase activity combined with reduced glutathione levels indicates increased sensitivity to damage from oxidative stress [63].

Other markers we describe are Advanced Oxidation Protein Products (AOPPs), nitrotyrosine (NT), and iNOS. AOPPs are formed as a result of the exposure of plasma proteins to free radicals [64]. A study by Simoncini et al. showed that AOPP levels in patients with FD are significantly elevated compared to the control group. The presence of AOPP has also been demonstrated in both patients treated with enzyme replacement therapy and in previously untreated patients [30]. Studies indicate that 3-nitrotyrosine (3-NT) may be a marker of oxidative stress. The pathogenesis highlights the role of nitric oxide, which reacts with tyrosine residues, nitrating them to 3-NT [65]. A 6-fold higher concentration of 3-NT has been demonstrated in untreated FD patients compared to treated patients, indicating the possible usefulness of measuring 3-NT concentration [66]. Patients with cardiomyopathy in the course of FD also showed a significant increase in iNOS and NT. In a study by Lee et al. on cardiomyocytes from patients with FD, an increase in iNOS concentration was observed in cells before the onset of pathological inclusions [41]. The above studies indicate the presence of oxidative stress and the potential usefulness of AOPP, 3-NT, and iNOS as biomarkers in monitoring the course and treatment of FD.

Table 1 summarizes the characteristics of oxidative stress biomarkers observed in Fabry disease.

## 4. Organ-Specific Clinical Consequences

The organ-specific clinical manifestations of FD arise from the shared mechanisms detailed in Section 2. Below, we focus on how these pathways translate into distinctive cardiac, renal and neurological phenotypes.

### 4.1. Cardiovascular System

Fabry disease is associated with numerous clinical complications. One of these is cardiac dysfunction, the most characteristic manifestation of which is left ventricular (LV) hypertrophy, which remains insufficiently recognized in clinical practice. As a result, this condition frequently contributes to the development of heart failure as well as ventricular arrhythmias [69]. Mitochondrial dysfunction induced by oxidative stress, together with endothelial remodeling, described in Section 2, are mechanisms responsible for the impairment of LV functional capacity [19]. The progression of FD within the heart is gradual. Cardiomyocyte hypertrophy ultimately leads to cellular overload and, consequently, to cell death, which promotes the development of fibrosis [70]. LV hypertrophy is one of the principal parameters of FD and occurs relatively frequently in the affected population, being present in up to 50% of men and 33% of women with FD. Moreover, in individuals with unexplained LV hypertrophy, the gene responsible for the development of FD is detectable in approximately 4% of cases. Hypertrophy may involve not only the LV but also the right ventricle, which may subsequently lead to its dilation [71]. X-linked inheritance in FD results in a severe disease course being typically attributed to hemizygous males. In women, the disease usually presents with a milder phenotype and a later onset of symptoms compared with men. The heterogeneous clinical presentation of FD in females is attributed to the pattern of X-chromosome inactivation, a random event occurring during embryonic development. During this process, one of the X chromosomes is silenced, leading to mosaic expression of X-linked genes across different tissues in the female organism. The predominantly expressed allele (i.e., the mutant or the wild-type allele) influences not only the clinical course of the disease but also the selection of appropriate therapeutic strategies [72]. Late gadolinium enhancement reveals myocardial fibrosis and is useful in the diagnosis of a specific type of cardiomyopathy in Fabry disease. Loss of myocardial function and fibrosis of cardiac structures in the absence of left ventricular hypertrophy may affect approximately 25% of women with Fabry disease, as demonstrated by studies using late gadolinium enhancement. In 75% of patients with Fabry disease, replacement fibrosis involves the basal and mid-ventricular segments of the inferolateral wall of the heart [73,74]. Studies have demonstrated that oxidative stress is actively present in FD and correlates with cardiac changes, but direct relationship has not been established [5]. Additionally, it has been suggested that glycosphingolipid accumulation contributes to disturbances in ionic homeostasis through its effects on sodium and calcium channels, thereby promoting the activation of hypertrophic genes and conduction abnormalities. These changes are reflected on electrocardiography as bradycardia, shortening of the PR interval, and atrioventricular conduction disturbances. Furthermore, this disease is associated with an increased risk of both ventricular and supraventricular arrhythmias [40]. Cardiomyocytes in patients with FD are characterized by an increased frequency of spontaneous action potentials and a shortened action potential duration, while cells of the cardiac conduction system exhibit accelerated impulse conduction and prolonged refractory periods [75]. A systematic review by Vijapurapu et al. demonstrated that atrial fibrillation occurs in approximately 12% of patients with FD, and that about 10% of patients require pacemaker implantation. However, the authors emphasize that the true prevalence of conduction disorders may be higher, as many arrhythmias may remain undiagnosed due to their asymptomatic nature or failure to be captured during diagnostic testing [76]. The literature indicates that with increasing age, more severe complications tend to occur, most commonly in the form of right bundle branch block or left anterior fascicular block. In contrast, third-degree atrioventricular block is estimated to occur in approximately 12.7% of men and 1.6% of women [69]. Another systematic review conducted by Baig et al. included a total of 4185 patients with FD. Following their analysis, the authors concluded that the most common cause of death among these patients is related to cardiac complications, particularly sudden cardiac death; however, they emphasize the heterogeneity of the analyzed cohorts and the lack of studies focusing directly on sudden cardiac death as a primary endpoint [77]. Another important clinical issue is ischemic heart disease, which may coexist with FD. Reviews of studies indicate that myocardial ischemia is largely associated with coronary microvascular dysfunction, as evidenced by slow coronary flow, perfusion defects, reduced coronary reserve, and the absence of significant stenoses on coronary angiography. The latter finding suggests that this is not typical coronary atherosclerosis, although it does not preclude the presence of classic chronic coronary syndrome, as patients often present with risk factors that predispose them to conventional atherosclerosis [78,79]. Data from the Fabry Registry indicate that approximately 5.8% of middle-aged men and 3.7% of middle-aged women with FD experienced major cardiac events, frequently in the form of myocardial infarction [69]. Another significant clinical complication of FD is valvular heart disease, with a reported prevalence of 15–25%. The most commonly observed findings are mild regurgitation (typically involving the mitral and/or aortic valves) and leaflet thickening. However, reports also describe more severe forms presenting as severe aortic stenosis requiring surgical intervention [80,81].

### 4.2. Renal System

FD may also involve the kidneys, which occurs in approximately 55% of patients. Unfortunately, nephropathy in individuals with FD is often overlooked, resulting in underdiagnosis [15,77]. From the perspective of clinical manifestation, FD nephropathy can be primarily categorized into proteinuria, arterial hypertension, and progressive chronic kidney disease. In FD, podocytes are key cells that are particularly susceptible to injury [15]. In addition to being damaged, podocytes in FD detach from the glomerular basement membrane, which consequently leads to podocyturia. Excessive loss of podocytes into the urine disrupts the function of the glomerular filtration barrier. As a result of this process, irreversible structural changes may occur within the glomerulus, which can precede the development of proteinuria. The literature indicates that when approximately 60–80% of podocytes within a glomerulus are lost, glomerular function is impaired due to its gradual atrophy. Podocyte loss is clinically relevant, as it often heralds the onset of proteinuria [82]. Moreover, the severity of podocyturia correlates positively with the degree of proteinuria. Although an excessive number of podocytes in the urine could potentially serve as a marker of glomerulopathy, the lack of methodological standardization and limited availability prevent its routine use in clinical practice. With regard to proteinuria, it is a relatively common finding, affecting on average 85% of men as early as 14 years of age. Its prevalence and severity are lower in women, regardless of age or stage of chronic kidney disease [83]. Because proteinuria and hyperfiltration occur in the early phases of the disease, patients with FD are recommended to undergo annual direct measurements of glomerular filtration rate (GFR) in order to accurately assess renal function. High-grade proteinuria usually indicates the presence of chronic kidney disease, which may progress to end-stage renal failure [84]. Proteinuria itself is a useful and commonly employed marker of FD nephropathy, providing information on disease severity as well as treatment effectiveness. A more precise measure than total proteinuria is the assessment of albuminuria, as it occurs only when podocytes are damaged. Additionally, hematuria may be detected in patients with FD, although it is relatively uncommon [85]. Another important issue is blood pressure, which in patients with FD usually reaches lower values. The exact causes of this phenomenon are not fully understood; however, a hypothesis suggests autonomic dysfunction, likely caused by glycosphingolipid accumulation in nervous tissue and in the smooth muscle of blood vessels supplying these nerves. The latter process is referred to as vasculopathy and is characterized by smooth muscle hypertrophy. Nevertheless, as the disease progresses, hypertension develops, particularly with advancing chronic kidney disease and vascular involvement [86,87]. The literature also reports rarer tubular clinical manifestations of FD, such as distal renal tubular acidosis, isosthenuria, nephrogenic diabetes insipidus, and Fanconi syndrome [84]. Additionally, renal cyst formation has been observed in FD, although its pathogenesis has not yet been elucidated [87].

### 4.3. Nervous System

Patients diagnosed with FD are at risk of developing and experiencing progression of neurological symptoms. The main manifestations include symptoms of peripheral nerve damage as well as vascular injury of the brain [80]. Mechanisms mentioned in Section 2 are responsible for nerve damage, disruption of ion channel function, and dysfunction of the dorsal root ganglia. Changes in the peripheral nervous system (PNS) usually manifest as severe neuropathic pain and the aforementioned autonomic dysfunction, which, in addition to vascular abnormalities, is characterized by hypohidrosis, impaired intestinal peristalsis, pupillary constriction abnormalities, reduced tear and saliva production, sensory deficits, Raynaud-like symptoms, and, in later stages of the disease, orthostatic hypotension [10,88]. The cause of neuropathic pain may be small fiber neuropathy. The literature distinguishes two types of pain. The first type, also referred to as “Fabry crises,” is characterized by sudden, severe pain in the limbs radiating centrally. The second type of pain is chronic, also known as acroparesthesias, and manifests as burning, stabbing sensations or dysesthesia in the distal parts of the limbs [89]. Neuropathic pain has been reported in 60–80% of boys and in 40–60% of girls, with symptoms in females typically appearing several years later. Neuropathic changes affecting small fibers can be observed in unmyelinated C fibers, thinly myelinated Aδ fibers, and unmyelinated autonomic fibers. With advancing age, pain tends to diminish, which is attributed to nerve degeneration. Moreover, sensory neuropathy may lead to progressive numbness and reduced pain sensitivity [88]. Evidence regarding the involvement of large myelinated fibers in FD is inconclusive. Some sources report that large fiber involvement occurs in advanced stages of the disease [90], whereas others indicate that such changes are not observed in patients with FD [91,92]. In FD, the central nervous system (CNS) is also affected. Relatively common clinical manifestations include ischemic stroke and transient ischemic attacks (TIA), although the literature also reports cases of intracerebral hemorrhage, microbleeds, depression, and cognitive impairment [93]. The pathomechanism of stroke development in individuals with FD is associated both with conditions secondary to FD, such as valvular heart disease, ischemic heart disease, arrhythmias, hypertension, and renal failure, as well as with direct vascular pathology within the CNS involving both small and large vessels [94]. Data from the Fabry Registry, including 2446 patients, indicate that the prevalence of stroke was 6.9% in men and 4.3% in women, with the majority being ischemic strokes (86.8%) [88]. In a cross-sectional study by Natale et al. including a total of 40 patients, ischemic stroke occurred in 4 individuals (10%), of whom one also experienced multiple episodes of TIA [95]. Another clinical manifestation comprises white matter lesions (WML). The distribution of these lesions is described as nonspecific and multifocal. Their prevalence is approximately 45% and correlates positively with age and stroke risk. WML develop significantly faster in men than in women. WML have the potential to become a biomarker for detecting CNS involvement; however, further studies are required to standardize this marker before it can be implemented in routine clinical practice [96]. There are also reports of potential psychiatric symptoms in patients with FD, which, in addition to depression, include acute psychotic episodes, personality changes, and sleep disturbances [97].

### 4.4. Skin and Ocular System

The skin is another organ involved in the disease process of FD. Cutaneous manifestations are very common and clinically significant findings, and their presence may suggest the diagnosis of FD to clinicians. Dermatologic manifestations primarily include sweating abnormalities, angiokeratomas, telangiectasias, and lymphatic edema [98]. A systematic review by Al-Chaer et al., which analyzed data from multiple studies, demonstrated that the most frequent dermatologic abnormalities were sweating disturbances, affecting 57.6% of patients, followed by angiokeratomas (51.5%); telangiectasias, lymphatic edema, and hair abnormalities were observed in 19.9% of the 2396 patients with reported skin manifestations [99].

Ocular manifestations, similar to cutaneous findings, are of substantial value in the diagnostic process of FD. The main ocular abnormalities observed in FD include corneal and lens opacities as well as vascular changes [100]. The most characteristic hallmark of FD is keratopathy, known as cornea verticillata. It is characterized by the presence of pigmented deposits within the cornea, predominantly in its inferior one-third. This finding may represent one of the earliest manifestations of the disease, appearing in affected individuals as early as 4 years of age. Disease severity correlates positively with the degree of corneal pigmentation [101]. Patients with FD also exhibit conjunctival vessel tortuosity and aneurysm formation [102].

## 5. Treatment

### 5.1. Established Clinical Therapies

A current cornerstone method of treatment is enzyme replacement therapy, as it directly targets the fundamental cause of Fabry disease, being lack of alpha-galactosidase A. The treatment aims to replace the lacking enzyme and slow the disease progression [103]. Nowadays, several ERT options are available. Agalsidase alfa and beta have been the mainstay for almost 20 years. Both agalsidase alfa and agalsidase beta are recombinant forms of human α-galactosidase A used as ERT for Fabry disease. Their mechanism of action is fundamentally the same: both enzymes supplement the deficient or absent endogenous α-galactosidase A, facilitating the breakdown of Gb3 in lysosomes and reducing its pathological accumulation [104]. Agalsidase alfa has been shown to stabilize or improve clinical parameters important in Fabry disease, such as pain reduction, decrease in left ventricular mass and maintenance of renal function in both male and female patients [105,106]. Long term studies demonstrate sustained benefits in cardiac and renal outcomes, as well as improved quality of life and reduced disease burden [107]. Agalsidase alfa is generally safe, with a low incidence of serious adverse events and infusion reactions [108]. Long-term studies concerning agalsidase beta show that early initiation of agalsidase beta slows the progression of renal, cardiac, and cerebrovascular complications, with the greatest benefit seen in patients who start treatment at a younger age and with less advanced disease [109,110]. In children, agalsidase beta improves pain, gastrointestinal symptoms, and quality of life, and may prevent serious organ involvement if started early [111]. Most research finds no significant difference in clinical outcomes between both types of agalsidase, though some biochemical and immunogenicity differences exist. Large cohort and randomized studies show no significant difference in major clinical event rates (renal, cardiac, cerebrovascular) between agalsidase alfa and beta at their approved doses [109,112]. Both therapies stabilize renal function and cardiac structure. Some studies report a greater reduction in left ventricular mass and plasma lyso-Gb3 with agalsidase beta, especially in men with classical Fabry disease, though this does not translate into clear clinical superiority [113]. Switching from beta to alfa (which is often due to supply shortages) in general results in stable organ function and quality of life; however, some studies note a rise in lyso-Gb3 and clinical events [114]. Agalsidase beta has a higher mannose-6-phosphate and sialic acid content, thus leading to greater cellular uptake in some tissues, but it does not translate to major clinical differences. Beta is also associated with higher risk of anti-drug antibody development which may affect biochemical response, but same as with factors mentioned above, no significant clinical outcome differences have been yet reported [112,115]. While agalsidases are generally safe and effective, there are some key limitations. First one is that both alfa and beta are cleared quickly from circulation, requiring frequent (biweekly) infusions and leading to fluctuating enzyme levels. Short half-life may limit tissue uptake and therapeutic efficacy, especially in organs with slow enzyme turnover [11,116]. Another important issue is immunogenicity and anti-drug antibodies. Studies show that up to 40% male patients develop neutralizing antibodies, which can reduce ERT effectiveness and are often persistent for years. What is more, cross-reactivity of anti-drug antibodies between alfa and beta limits the benefit of switching between these therapies [115]. A newer alternative for above medications is pegunigalsidase alfa. It is a PEGylated recombinant α-galactosidase A enzyme. Its design aims to address limitations of earlier enzyme replacement therapies. Pegunigalsidase alfa is chemically modified (PEGylated) and produced in plant cells, resulting in a prolonged plasma half-life (up to ~80–120 h) compared to agalsidase alfa and beta (≤2 h) [117,118]. The PEGylation is carried out to mask immunogenic epitopes, leading to lower rates of anti-drug antibody formation and milder infusion reactions compared to other ERTs [119]. Phase 3 trials (BALANCE, BRIDGE, BRIGHT) show pegunigalsidase alfa is non-inferior to agalsidase beta in slowing renal decline (eGFR slope difference: −0.36 mL/min/1.73 m^2^/year) and stabilizes cardiac function [117,120]. Most adverse events are mild or moderate. Infusion-related reactions and ADA rates are lower than with agalsidase beta. Patients switching from agalsidase alfa or beta to pegunigalsidase alfa maintain or improve renal function and show reduced lyso-Gb3 levels [119,120,121]. Another important advantage is extended dosing interval. Due to long half-life, pegunigalsidase alfa can be administered every 2 or 4 weeks, potentially reducing treatment burden and improving adherence [122]. Chaperone therapy, specifically with the oral agent migalastat, represents a significant advance in Fabry disease treatment. Unlike ERT, chaperone therapy is designed for patients with specific “amenable” GLA gene mutations, offering a targeted and convenient oral alternative. Amenability is defined by a validated in vitro assay using HEK293 cells expressing the patient’s GLA variant. A mutation is considered amenable if, in the presence of 10 μmol/L migalastat, α-galactosidase A activity increases by ≥1.2-fold over baseline and by an absolute ≥ 3% of wild-type activity [123,124]. Most amenable mutations are missense mutations. Large deletions, frameshift, nonsense (except near the C-terminus), and splicing mutations are generally non-amenable [124]. Frequently reported amenable mutations include p.N215S, p.A143T, R363C, M296I, L300P, R301Q, G104V, R112H, D136E, and L166G. About 35–50% of Fabry patients have amenable mutations, but the exact list is continually updated as new variants are tested [11,123,124,125]. Migalstat is a small-molecule iminosugar, that reversibly binds to the active side of amenable mutant α-galactosidase A in the endoplasmic reticulum stabilizing its structure and preventing premature degradation [126]. The stabilized enzyme-migalastat complex is transported from the ER to the lysosome. In the acidic lysosomal environment, migalastat dissociates, freeing the enzyme to function normally. Once released, the enzyme can break down Gb3 and related substrates, reducing their pathological accumulation in cells [11,123,124]. In the pivotal ATTRACT phase III trial of ERT-experienced adults, migalastat and ERT had similar preservation of renal function over 18 months, while migalastat produced a significant reduction in left-ventricular mass index (LVMi) and numerically fewer renal/cardiac/cerebrovascular events [127,128]. A large SLR and narrative reviews conclude that migalastat generally matches ERT for renal outcomes and offers clear cardiac benefit, with a comparable safety profile [123,129]. Long-term trial follow-up (up to ~8.6 years) shows stable eGFR and low incidence of serious renal, cardiac, and cerebrovascular events (≈48 events/1000 patient-years overall) [130,131]. Real-world multicenter and registry data (FAMOUS, followME) show modest declines in eGFR typical of disease course, with consistent reductions in LVMi over 1–4 years [132,133]. In adolescents with amenable variants, 4-year follow-up shows normal/stable kidney and heart parameters, stable lyso-Gb3, and improved exertional pain, with good tolerability. Main restrictive factor is that benefit is restricted to amenable mutations; misclassification can lead to poor biochemical and clinical response. Some data analyses suggest disease progression in a subset after switching from ERT, therefore patients must be closely monitored [134,135,136]. Table 2 summarizes established clinical treatment options for Fabry disease, comparing enzyme replacement therapy (ERT) with chaperone therapy.

### 5.2. Supportive and Life-Style Based Management

Supportive management plays an important role in the holistic care of FD. Non-pharmacological approaches include diet modification, physical activity, pain rehabilitation as well as psychological support. Nutritional management in FD focuses on reducing renal and gastrointestinal strain. A low-sodium diet is recommended in prevention of hypertension and limiting proteinuria, which accelerates renal decline [138]. Sodium restriction enhances the efficacy of renoprotective medications, such as ACEI and ARBs. Adequate hydration and avoidance of excessive protein intake may further help protect kidney function. Fabry disease causes intestinal dysbiosis and disturbances in bacterial metabolites, including short-chain fatty acids and tryptophan catabolites [39]. The disease metabolite, lyso-Gb3, itself modulates the composition of the microbiota, promoting the growth of pro-inflammatory bacteria and reducing butyrate production, which may exacerbate intestinal symptoms and inflammation [39]. In this context, diet, bioactive food components and microbiota-targeted interventions are seen as potential adjuncts to treatment, capable of modifying inflammation, oxidative stress and gastrointestinal symptoms in Fabry disease [139]. In case of gastrointestinal manifestations like postprandial pain, diarrhea or bloating—a low FODMAP diet can significantly reduce symptoms by limiting fermentable carbohydrates that contribute to dysbiosis and malabsorption [140]. In addition, probiotic and prebiotic supplementation may restore gut microbiota balance and alleviate chronic gastrointestinal discomfort [141]. Since deficiencies in vitamin D and antioxidants have been correlated with cardiac hypertrophy, disfunction of the endothelium and inflammation in FD, supplementation with vitamin D and polyphenol-rich foods is recommended to counter oxidative stress [142,143]. Soy isoflavones, mainly genistein and daidzein, improve key components of metabolic syndrome—obesity, dyslipidemia, hypertension, and hyperglycemia-through estrogen receptor signaling, PPAR activation, AMPK stimulation, and anti-inflammatory/antioxidant actions [144,145]. They reduce visceral adiposity, improve lipid profiles, and ameliorate insulin resistance and adipose-tissue inflammation in animal and human studies [146,147,148]. In NAFLD, soy isoflavones lower triglycerides, LDL-C, total cholesterol, waist/hip circumference and improve hepatic steatosis when added to standard lifestyle advice [146,147]. Similar lipid-lowering and cardioprotective effects are reported in type 2 diabetes and broader metabolic syndrome populations [149]. In Fabry disease, polyphenols are highlighted for their potential to modulate inflammation and oxidative stress-central mechanisms in organ damage-supporting the use of antioxidant, anti-inflammatory dietary patterns as complements to enzyme or chaperone therapy [143]. Although isoflavones are not specifically tested in Fabry disease, they fit this polyphenol class. Physical activity and rehabilitation are equally important in supportive management. Exercise intolerance is common in Fabry disease, with average VO_2_max reaching only 78.8% of predicted values compared with age-matched healthy individuals [150]. Despite these limitations, moderate aerobic exercise (30–40 min, 3 times weekly) has been associated with a 15–20% increase in exercise tolerance and improved fatigue scores over 12 weeks [143]. Supervised exercise also enhances endothelial function and cardiac performance, if overheating and dehydration are avoided. Physical and occupational therapy programs help preserve mobility, joint flexibility, and fine motor coordination while reducing fatigue and preventing musculoskeletal deconditioning. Pain management remains a central challenge in FD, as neuropathic pain affects up to 80% of men and 60% of women, frequently persisting despite ERT [151]. Integrative pain management combining pharmacological agents with cognitive-behavioral therapy, physiotherapy, and mindfulness-based interventions has been shown to reduce chronic pain intensity by 30–35% and improve health-related quality of life by 0.1–0.2 EQ-5D points [152]. Participation in multidisciplinary pain programs has also been associated with a 25% reduction in analgesic use over one year [151]. Approximately 40% of patients report significant depressive or anxiety symptoms, and nearly 25% experience chronic fatigue that interferes with daily life [153]. A pilot interventional study of adults with Fabry disease receiving 6 months of structured psychological counseling (in-person or tele-counseling) showed improvements in depression, mental health–related quality of life, adaptive functioning, and subjective pain, sustained 6 months after counseling ended [154]. A qualitative study of adults with Fabry disease found patient associations and peer groups are among the most valued coping resources: they reduce loneliness, provide emotional support, facilitate information sharing, and help with disease acceptance and normalization [155].

### 5.3. Future Targeted Redox-Modulating Therapies

Therapeutic strategies for FD are under continuous exploration, with the aim of identifying more effective, more specific, and pathway-targeted treatment modalities. A multimodal approach, integrating established strategies with novel and experimental interventions, represents the future of effective patient management [3].

The use of autologous hematopoietic stem cells (HSCs) as a gene therapy platform employing lentiviral vectors also presents promising results for future clinical application. Early-phase trials have confirmed both the safety and efficacy of this approach. The FACTs study reported five-year follow-up data demonstrating sustained expression and activity of α-galactosidase A (α-Gal A) in peripheral blood cells, accompanied by durable reductions in plasma lyso-Gb3 levels and stabilization of renal function. This strategy offers substantial potential as a renewable source of enzymatic activity, as genetically corrected cells continuously produce and secrete functional enzyme, resulting in long-term reduction of oxidative stress biomarkers and stabilization of organ function without significant adverse events [156]. This represents a milestone in the development of gene therapy for Fabry disease, potentially eliminating the need for lifelong treatment. Nevertheless, further randomized studies involving larger patient cohorts and direct comparisons with established therapies such as ERT and emerging. Substrate Reduction Therapy (SRT)- a pharmacological approach aimed at inhibiting the synthesis of glycosphingolipids- are required. An increasing number of innovative therapeutic concepts are also being developed to address the complex pathologies associated with Fabry disease. Given that mitochondrial dysfunction represents a key component of FD pathogenesis, novel approaches focus on pharmacological support of mitochondrial homeostasis. Two compounds-polydatin (a natural polyphenol) and nicotinamide-have been investigated in combination therapy. This intervention resulted in increased levels of GFM1 transcripts and improved expression of the EF-G1 protein, as well as enhanced expression of respiratory chain complex proteins (I, II, IV, and V), encoded by both nuclear and mitochondrial DNA. These changes led to improved mitochondrial respiration and ATP production. Treatment restored mitochondrial membrane potential, reduced mitochondrial fragmentation, improved mitochondrial network morphology, and increased mitochondrial protein synthesis. Additionally, activation of key mitochondrial Unfolded Protein Response (mtUPR) pathways and the mitochondrial deacetylase Sirtuin 3 (SIRT3), a critical component of cellular protective mechanisms, was observed [157].

Another mitochondria-focused therapeutic strategy targets autophagy, a process known to be dysregulated in Fabry disease. Excessive accumulation of lipid substrates due to impaired autophagy disrupts intracellular homeostasis. Agents such as rapamycin and other mechanistic Target of Rapamycin (mTOR) kinase modulators may counteract this pathology and represent promising therapeutic options. However, further studies are required to fully explore and refine this treatment approach [158,159].

Moreover, reduced oxidative phosphorylation capacity and dysregulation of microRNAs that directly influence mitochondrial function represent additional therapeutic targets and form another branch of emerging innovative treatments in Fabry disease [20]. Interventions such as lomerizine, a calcium channel blocker, have demonstrated multidimensional benefits in Fabry disease models by reducing mitochondrial calcium overload and ROS production while simultaneously inhibiting proangiogenic inhibitors and promoting endothelial nitric oxide synthase expression. In vivo studies using murine models of Fabry disease have shown that oral lomerizine administration attenuated left ventricular hypertrophy, renal fibrosis, anhidrosis, and heat intolerance. These findings suggest that dysregulated mitochondrial calcium handling may represent a central mechanism underlying multiple manifestations of Fabry disease and a viable therapeutic target [160]. One of the complications of Fabry disease is nephropathy, caused by excessive accumulation of lyso-Gb3, leading to podocyte injury and induction of cell death via a Receptor-Interacting Serine/Threonine-Protein Kinase 3 (RIPK3) dependent regulated necrosis pathway. This represents an alternative mechanism of glomerular destruction independent of classical apoptosis. Understanding this pathway provides a potential target for adjunctive therapies independent of enzymatic defect correction, such as intervention in the RIPK3 pathway through the use of specific inhibitors [161]. Although promising, this therapeutic concept requires further investigation to clarify its mechanisms and confirm efficacy prior to clinical validation. Another therapeutic avenue involves targeting endothelial dysregulation, which leads to endothelial dysfunction and subsequent complications such as left ventricular hypertrophy (LVH). The pathogenesis of these changes is based on elevated levels of pro-inflammatory cytokines and oxidative stress-related proteins. The development of new therapeutic strategies focuses on modulation of endothelial-associated oxidative stress pathways, representing a promising direction for the prevention and treatment of cardiovascular complications in Fabry disease [5]. Inhibition of the Rho kinase pathway may counteract pathological remodeling of the cardiovascular and renal systems induced, in part, by oxidative stress. Rho kinase inhibitors such as Y-27632 have demonstrated the ability to reduce oxidative stress signaling, suppress pro-inflammatory mediators, and attenuate vascular changes induced by hemodynamic stress. Emerging evidence suggests that next-generation Rho kinase inhibitors with improved selectivity profiles, such as Y-33075, may provide enhanced therapeutic efficacy by reducing both inflammation and oxidative stress while promoting protective cellular responses. Although this approach has not yet been specifically studied in Fabry disease, it represents a pioneering strategy for treating pathologies also present in FD and may translate into future clinical benefit [162]. Large-scale clinical studies are required to achieve full validation of this approach.

## 6. Discussion

Oxidative stress is now recognized as a central, integrative mechanism in Fabry disease. Glycosphingolipid accumulation (Gb3, lyso Gb3) in lysosomes triggers excess ROS, impaired antioxidant defenses (reduced glutathione, thiols, HO-1) and nitric oxide synthase dysfunction, promoting endothelial damage and vascular inflammation [5,30,163]. This redox imbalance drives cardiovascular renal remodeling, myocardial hypertrophy, arrhythmias and nephropathy, and persists despite enzyme replacement therapy, helping explain ongoing organ damage in late treated patients [5,6]. At the cellular level, oxidative stress intersects with mitochondrial dysfunction, defective autophagy and apoptosis in cardiomyocytes, podocytes and tubular cells, amplifying fibrosis and cell loss [62,164,165]. Consequently, antioxidants (e.g., glutathione repletion, green tea polyphenols) and nutrition based strategies are emerging as promising adjuncts to disease specific therapies [55,139,164].

Molecular pathways in Fabry disease converge on a persistent redox imbalance-driven himby glycosphingolipid storage. Accumulation of Gb3 and lyso Gb3 activates NADPH oxidases (p22phox upregulation) and enhances mitochondrial ROS generation, while antioxidant systems such as heme oxygenase 1, glutathione and total antioxidant capacity are reduced [5,61,158]. This shift promotes lipid and protein oxidation (MDA, AOPP, carbonyls), NO inactivation and endothelial dysfunction, creating a pro-vasoconstrictive, pro-thrombotic phenotype with increased adhesion molecules and renin–angiotensin activation [4,5,30].

ROS excess triggers redox sensitive signaling, including RhoA/ROCK (MYPT 1 phosphorylation), NF-κB and TGF-β pathways, which drive vascular and cardio renal remodeling and fibrosis [9,158]. Transcriptomic and proteomic studies in Fabry podocytes and GLA deficient zebrafish kidneys show altered expression of genes and proteins linked to oxidative stress, mitochondrial metabolism, ER stress, apoptosis, autophagy and extracellular matrix homeostasis, indicating that disturbed redox signaling becomes partly autonomous from Gb3 storage [166,167].

In vitro, Gb3 directly perturbs electron transport chain complexes and increases reactive species, confirming a primary mitochondrial contribution to oxidative stress [168]. Importantly, redox abnormalities persist in patients on enzyme replacement therapy, suggesting that oxidative stress is a parallel, therapeutically relevant axis of Fabry pathophysiology rather than a mere byproduct of storage [6,61,158].

Clinical manifestations of Fabry disease reflect organ specific consequences of chronic oxidative and nitrosative stress. Elevated plasma malondialdehyde (MDA; ~55 vs. 30 nmol/mL) in Fabry patients links lipid peroxidation with LV hypertrophy and cardiovascular renal remodeling [9]. Increased 3 nitrotyrosine and iNOS in myocardium and plasma document nitric oxide–derived reactive species and correlate with extensive cardiomyocyte DNA damage (8-OHdG positivity in 25% of nuclei) and a 187 fold rise in apoptosis, providing a mechanistic bridge to fibrosis, LV dysfunction and arrhythmias [4,7,169].

In the kidney, oxidative stress is associated with progressive Fabry nephropathy. Higher MDA and p22phox expression, together with reduced Heme Oxygenase-1 (HO-1), accompany proteinuria and declining eGFR, implicating ROS driven injury in glomerulosclerosis and tubulointerstitial fibrosis [9]. Podocyturia appears early and correlates with albuminuria and lower GFR, indicating that glomerular damage precedes overt renal failure and may be aggravated by oxidative mechanisms [87,170].

Oxidative stress biomarkers such as AOPP, 8-OHdG and 3-nitrotyrosine therefore serve as translational tools, connecting subcellular redox imbalance to clinically relevant endpoints across heart, kidney and vasculature [4,9,30,169]. Their partial or absent normalization under enzyme replacement therapy underscores a residual redox burden and supports exploring antioxidant or redox modulating adjuncts to prevent late organ damage in Fabry disease [9,30,160].

Fabry disease requires therapeutic strategies that go beyond simply “clearing” Gb3/lyso Gb3, as current treatments only partially inhibit organ progression and do not reverse fibrosis. Enzymatic replacement therapy (ERT; agalsidase alfa/beta, newer pegunigalsidase alfa) regularly reduces storage biomarkers and stabilizes kidney and heart function, especially when implemented in the early stages of the disease [103,116,136]. Limitations include a short half-life, the need for intravenous infusions every 2 weeks, incomplete tissue penetration (brain, heart muscle in late stages), and the production of neutralizing antibodies in approximately 40% of men, which may weaken the clinical effect and maintain the redox burden [11,115,126]. Pegunigalsidase alfa prolongs circulation time and appears to be less immunogenic, but its long-term benefits over classic preparations remain uncertain [116,137].

Migalastat, an oral chaperone, offers stabilization of endogenous α Gal A and similar efficacy to ERT in terms of renal function and left ventricular mass, but is only available to ~35–50% of patients with amenable mutations and does not reverse existing organ damage [123,124,171].

Newer approaches—substrate reduction therapy (lucerastat, venglustat), ex vivo/in vivo gene therapy, mRNA ERT and ‘second-generation’ ERT—show promising decreases in lyso Gb3 and a sustained increase in enzyme activity, but remain in the early stages of research, with no evidence of complete normalization of oxidative stress or reversal of fibrosis [116,137,171,172].

Despite growing data on oxidative stress in Fabry disease, there is still a lack of causal evidence that ROS and antioxidant deficiencies directly drive cardiac, renal and vascular remodeling—most studies are observational or preclinical, without interventions targeting redox with hard endpoints [4,173,174].

Biomarkers are a significant limitation. Classic markers of oxidative stress, such as MDA or AOPP, are organ-nonspecific and reflect generalized damage rather than cardiac or renal prognosis [4,31,175]. Podocyturia, although promising as an early marker of nephropathy, remains poorly validated prognostically. In turn, proteomic signatures of inflammation and angiogenesis (e.g., FGF2, Vascular Endothelial Growth Factor (VEGF), Interleukin-7 (IL-7)) provide information orthogonal to lyso Gb3, but are not yet linked to hard clinical events or treatment response [175]. Metabolomics in Fabry mice reveals abnormalities in glutathione, NO and kynurenine pathways, but the translation of these panels to humans is only just beginning [176].

Prospective cohorts combining proteomics, metabolomics and classic risk factors to build multi-marker risk stratification models, similar to large oncology and cardiology studies, are a priority [177,178]. Randomized trials of antioxidants and autophagy modulators as adjuncts to ERT/chaperones are also needed, with biomarker panels (redox, inflammation, angiogenesis) to monitor response [4,173]. Translational models—mice with symptoms and iPSC lines with different genotypes—should be used to define ‘therapeutic windows’ and study sex differences in antioxidant response [173,175,176].

## 7. Conclusions

This review underscores the pivotal role of oxidative stress in the pathophysiology of Fabry disease, arising as a downstream consequence of glycosphingolipid (Gb3 and lyso-Gb3) accumulation. This process contributes to mitochondrial dysfunction, activation of NADPH oxidases, impairment of antioxidant defenses, and initiation of inflammatory pathways, ultimately leading to multi-organ involvement, including cardiac (left ventricular hypertrophy, arrhythmias), renal (proteinuria, progressive nephropathy), and neurological (neuropathic pain, cerebrovascular events) manifestations.

The available evidence demonstrates increased levels of oxidative stress biomarkers-such as MDA, 8-OHdG, AOPP, and 3-nitrotyrosine-in both treated and untreated patients, indicating that oxidative damage persists independently of enzyme replacement therapy. These findings support the potential value of oxidative stress markers in risk assessment and highlight antioxidant strategies as promising adjunctive therapeutic approaches. Future research should prioritize randomized clinical trials evaluating oxidative stress-targeted interventions, the validation of organ-specific biomarkers, and translational studies aimed at clarifying genotype–phenotype discrepancies.

## 8. Future Directions

### 8.1. Knowledge Gaps and Future Research Perspectives

Despite recognition of oxidative stress as a key contributor to FD pathogenesis, its precise role in disease progression remains incompletely understood, even when lysosomal storage dysfunction is addressed [4,30,32,55,158]. Further research is required to clarify the clinical significance of oxidative stress, mitochondrial dysfunction, and associated inflammatory processes.

The persistence of oxidative stress despite ERT highlights unresolved questions regarding mitochondrial dysfunction, including the relevance of the mitochondrial unfolded protein response and dysregulation of mitochondria-associated microRNAs [20,32,179]. The relationship between mitochondrial stress and organ-specific manifestations has not been systematically characterized in long-term human studies, limiting the development of targeted interventions.

Uncertainties remain regarding the interplay between oxidative stress and inflammation, the impact on endothelial dysfunction, and the relative contributions of mitochondrial versus extra-mitochondrial ROS sources [180,181,182]. The timing and reversibility of oxidative stress-related tissue damage, as well as the mechanisms leading to irreversible fibrosis, are not fully defined [26,183].

Biomarker development faces multiple limitations. Existing oxidative stress markers, such as AOPP, MDA, total thiol groups, and lyso-Gb3, are not specific for organ involvement and lack validation for predicting disease progression or treatment response [30,184]. Candidate markers, including p22phox and cardiovascular biomarkers such as A Disintegrin And Metalloproteinase with Thrombospondin motifs-13 (ADAMTS-13), Tumor Necrosis Factor-alpha (TNF-α), Growth Differentiation Factor-15 (GDF-15), Vascular Endothelial Growth Factor A (VEGFA), Myeloperoxidase (MPO), and Monocyte Chemoattractant Protein-1 (MCP-1) remain under investigation [5,8]. Proteomic and metabolomic signatures show promise for patient stratification and monitoring, but clinical validation is still incomplete [20,51,175]. The optimal biomarkers for oxidative stress-modulating therapies, as well as assessment timing and patient selection, are yet to be established [29,179].

Additional gaps include differential tissue sensitivity to oxidative stress, unexplained heterogeneity in genotype–phenotype correlations, and the lack of standardized integration of oxidative stress profiles with clinical outcomes [68,152,185,186,187]. The role of endogenous antioxidant capacity and sex-specific differences remains poorly characterized, particularly regarding heterozygous female carriers [4,188].

Addressing these limitations requires systematic, interdisciplinary research to better define mechanisms, validate biomarkers, and inform precision medicine strategies for Fabry disease.

### 8.2. Translational Relevance and Clinical Implications

Translational research is central to understanding FD pathogenesis and its molecular alterations, enabling the identification of pathways for targeted therapeutic strategies [166]. Preclinical studies assessing oxidative stress-targeted interventions across cardiac, renal, and neurological systems can guide patient and organ selection for therapy and identify critical intervention windows [30,166]. Systematic translational frameworks are essential to bridge the gap between molecular insights and clinical application, particularly given the heterogeneity of FD and the need for mechanistic patient stratification based on oxidative stress, inflammation, mitochondrial dysfunction, and genotype–phenotype relationships [4,189].

Development of reliable biomarkers depends on translational research, which facilitates the identification of disease-specific molecular signatures reflecting activity and organ involvement. Proteomic, metabolomic, and transcriptomic approaches have revealed plasma protein changes and dysregulated mitochondria-associated microRNAs, supporting patient stratification and monitoring of therapy response [20,51]. Biomarkers such as protein carbonyls, 3-nitrotyrosine, and oxidative stress-related enzymes are under evaluation for their clinical applicability [30,169].

Translational studies also support the transfer of preclinical findings to human research. Early detection of mitochondrial dysfunction in animal models highlights potential intervention points prior to substrate accumulation [166]. Symptomatic transgenic mouse models allow for evaluation of oxidative stress-targeted therapies across multiple organs and assessment of surrogate endpoints prior to clinical trials [190]. Additionally, translational studies provide mechanistic insight into therapeutic transitions, such as from enzyme replacement therapy to oral migalastat, supporting quality-of-life improvements while maintaining metabolic control [191].

Collaborative networks and dedicated FD research centers integrating preclinical and clinical resources are crucial for advancing translational studies. International registries offer longitudinal clinical data combined with biomarker and treatment response information, supporting large-scale translational discoveries [192]. Incorporating patient-centered outcomes and long-term follow-up ensures that oxidative stress-targeted therapies achieve meaningful clinical benefits, including improved quality of life and reduced disease progression, beyond biomarker modulation alone [156,193].

## Figures and Tables

**Table 1:** Oxidative stress biomarkers in Fabry disease.

Biomarker	Sample Source	Clinical Relevance in Fabry Disease	Limitations	References
MDA	Plasma	Marker of lipid peroxidation;	Low specificity; influenced by diet and comorbidities	[4,6,9,30,56,67]
8-OHdG	Plasma, cardiomyocyte nuclei	Reflects oxidative DNA damage	High interindividual variability; lack of standardized cut-off values	[2,17,58,68]
AOPP	Plasma	Indicator of protein oxidation and systemic oxidative stress	Non-specific; elevated in multiple chronic diseases	[4,30,67]
3-NT	Plasma	Marker of nitrosative stress and endothelial dysfunction	Mainly experimental; limited clinical availability	[4,7]
GSH/GSSG	Plasma	Reflects cellular antioxidant capacity; altered in FD models	Influenced by metabolic and nutritional factors	[4,67]
iNOS	Cardiac and vascular tissues	Associated with nitrosative stress and myocardial injury	Requires tissue samples; not routinely measurable	[4,7]

**Table 2:** Summary of established clinical treatments for Fabry disease: ERT vs. Chaperone Therapy.

Feature	ERT	Migalastat	References
Mechanism of action	Exogenous α-Gal A replaces deficient enzyme	Stabilizes amenable mutant α-Gal A in lysosomes	[123,126,128,136,137]
Route & Frequency	Intravenous, every 2 weeks	Oral, every other day	[123,126,128,136,137]
Patient Eligibility	Any GLA mutation; amenability not required	Only migalastat-amenable GLA variants (≈35–50%)	[123,124,126,128,136,137]
Primary Clinical Impact	(Heart/Kidney)Stabilizes eGFR, cardiac structure; better if early	Maintains eGFR, often reduces left ventricular mass	[103,123,126,128,136,137]
Main Limitation	Lifelong IV infusions; infusion reactions, antibodies	Restricted to amenable variants; variable real-world response	[11,103,123,124,126,128,136,137]

## Data Availability

No new data were created or analyzed in this study. Data sharing is not applicable to this article.

## Abbreviations

The following abbreviations are used in this manuscript:

ADAMTS-13	A Disintegrin and Metalloproteinase with Thrombospondin motifs 13
AOPP	Advanced Oxidation Protein Products
CAT	Catalase
CNS	Central Nervous System
ERT	Enzyme Replacement Therapy
FD	Fabry Disease
FGF2	Fibroblast Growth Factor 2
GFR	Glomerular Filtration Rate
Gb3	Globotriaosylceramide
GSH	Reduced Glutathione
GSSG	Glutathione Disulfide
HO-1	Heme Oxygenase-1
ICAM-1	Intercellular Adhesion Molecule 1
iNOS	Inducible Nitric Oxide Synthase
iPSCs	Induced Pluripotent Stem Cells
LVMi	Left Ventricular Mass Index
Lyso-Gb3	Globotriaosylsphingosine
MAPK	Mitogen-Activated Protein Kinase
MCP-1	Monocyte Chemoattractant Protein-1
MDA	Malondialdehyde
MPO	Myeloperoxidase
mtUPR	Mitochondrial Unfolded Protein Response
NF-κB	Nuclear Factor Kappa B
NOX	NADPH Oxidases
NT	Nitrotyrosine
RIPK3	Receptor-Interacting Serine/Threonine-Protein Kinase 3
ROS	Reactive Oxygen Species
SIRT3	Sirtuin 3
SOD	Superoxide Dismutase
SRT	Stereotactic Radiotherapy
TNF-α	Tumor Necrosis Factor Alpha
VEGF	Vascular Endothelial Growth Factor
VEGFA	Vascular Endothelial Growth Factor A
VEGFR2	Vascular Endothelial Growth Factor Receptor 2
WML	White Matter Lesions
α-Gal A	α-Galactosidase A
α-SMA	Alpha-Smooth Muscle Actin
3-NT	3-Nitrotyrosine
4-HNE	4-Hydroxy-2-Nonenal
8-OHdG	8-Hydroxy-2′-Deoxyguanosine
